# Protocol for characterization of spatiotemporal network dynamics in cortical and hippocampal assembloids

**DOI:** 10.1016/j.xpro.2026.104463

**Published:** 2026-03-27

**Authors:** Asia R. Guzman, Colin M. McCrimmon, Daniel Toker, Bennett G. Novitch, Momoko Watanabe, Ranmal A. Samarasinghe

**Affiliations:** 1 Department of Neurology, University of California, Los Angeles, David Geffen School of Medicine, Los Angeles, CA 90095, USA; 2 Department of Neurobiology, University of California, Los Angeles, Los Angeles, CA 90095, USA; 3 Eli and Edythe Broad Center for Regenerative Medicine and Stem Cell Research, University of California, Los Angeles, Los Angeles, CA 90095, USA; 4 Intellectual Development and Disabilities Research Center, University of California, Los Angeles, Los Angeles, CA 90095, USA; 5 Department of Anatomy and Neurobiology, School of Medicine, University of California, Irvine, Irvine, CA, USA; 6 Sue & Bill Gross Stem Cell Research Center, School of Medicine, University of California, Irvine, Irvine, CA, USA

**Keywords:** Neuroscience, Stem Cells, Organoids

## Abstract

This protocol generates neural assembloids from human induced pluripotent stem cells (hiPSCs) to model human brain circuitry. It details the differentiation of excitatory-predominant hippocampal (Hc) and cortical (Cx) organoids and their fusion with inhibitory interneuron-predominant ganglionic eminence (GE) organoids. We then detail procedures for maintaining assembloids to promote interneuron migration and network integration, followed by functional assessment using two-photon calcium imaging to measure neuronal activity.

For complete details on the use and execution of this protocol, please refer to McCrimmon et al.^1^

## Highlights

• Techniques to generate unfused cortical, GE, and hippocampal organoids
• Key steps for generating hippocampal and cortical assembloids
• MATLAB-supported workflow for calcium-indicator imaging and network analysis

## Abstract (editor highlights)

Publisher’s note: Undertaking any experimental protocol requires adherence to local institutional guidelines for laboratory safety and ethics.

## Graphical abstract

**Figure:**
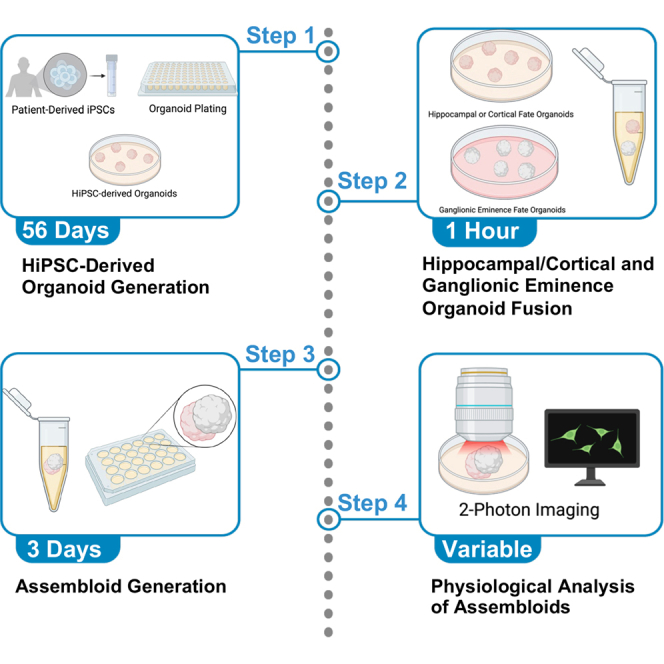

## Before you begin

Human-induced pluripotent stem cell (hiPSC)–derived organoids enable modeling of human-specific neuronal network properties not captured by animal systems. Hippocampal (Hc) and cortical (Cx) organoids typically lack significant numbers of inhibitory interneurons, limiting their physiological relevance. This protocol describes the generation of neural assembloids by fusing Hc or Cx organoids with ganglionic eminence (GE) organoids to introduce inhibitory components, promote interneuron migration, and enable functional circuit formation. It is important to confirm the identity of all cell lines prior to beginning. Though hiPSCs are commonly maintained on Matrigel-coated plates under feeder-free conditions, mouse embryonic fibroblast (MEF) layers were used in this protocol as they provide additional extracellular and paracrine support that promotes stable pluripotency and long-term undifferentiated growth.

### Innovation

This protocol builds upon established methods for generating cortical (Cx)+ganglionic eminence (GE) assembloids^2^ to generate hippocampal (Hc)+GE assembloids, which had not previously been described. By adapting fusion timing and patterning conditions to the hippocampal lineage, this approach enables interneuron migration and functional integration within developing hippocampal-like networks. The resulting Hc+GE assembloids display emergent patterns of oscillatory and synchronous activity,^1^ captured through a streamlined calcium-imaging workflow described here. Together, this protocol provides the first experimental framework to generate and functionally interrogate human hippocampal inhibitory–excitatory circuit assemblies in vitro.

#### Prepare hiPSC cultures

1. Maintain hiPSCs on mouse embryonic fibroblast (MEF) feeder layers in pluripotency medium to promote undifferentiated, long-term growth of iPSCs.
2. Verify pluripotency markers and overall culture health before initiating differentiation.

#### Generate organoids from hiPSCs

3. Aggregate hiPSCs into embryoid bodies (EBs) and initiate neural induction.
4. Transition EBs to neural induction medium to establish neuroectodermal identity.

#### Pattern organoids toward specific regional fates

5. For cortical (Cx) fate, inhibit TGFβ and WNT signaling to promote telencephalic identity.
6. For hippocampal (Hc) fate, supplement with dorsalizing factors to induce the medial pallium.
7. For ganglionic eminence (GE) fate, use ventralizing signals to generate interneuron progenitors.

#### Assemble organoids into neuronal assembloids

8. Fuse Hc or Cx organoids with GE organoids and maintain them in maturation medium.
9. Allow time for interneuron migration and integration into excitatory networks.

#### Assess neuronal network activity

10. Perform two-photon calcium imaging on mature assembloids to measure spontaneous neuronal activity.

### Institutional permissions

All of our experiments involving hiPSCs received prior approval from the University of California, Los Angeles (UCLA) Human Pluripotent Stem Cell Research Oversight (hpSCRO) Committee.

Note that prior to beginning any hiPSC work, you must obtain approval from your institution’s relevant committee or follow the rules of your local authorities.

## Key resources table

**Table:** 

REAGENT or RESOURCE	SOURCE	IDENTIFIER
**Antibodies**		

Glial fibrillary acidic protein (GFAP) 1:500	Abcam	Ab7260
Paired box 6 (Pax6) 1:1000	MBL	PD022
Forkhead Box G1 (FOXG1) 1:50	Abcam	Ab18259
BCL11B (Ctip2) 1:1000	Abcam	Ab18465
Special AT-Rich Sequence Binding Protein 2 (SATB2) 1:100	Abcam	Ab51502
Specificity Protein 8 (SP8) 1:100	Santa Cruz Biotechnology	SC-104661 (C18)
COUP-TF1(NR2F1) 1:100	Perseus Proteomics	PP-H8132
Lim Homeodomain 2 (LHX2) 1:100	Santa Cruz Biotechnology	Sc-517243
Prospero Related Homebox Gene 1 (Prox1) 1:200	EMD Millipore Corporations	MAB5654
Somatostatin (SST) 1:100	EMD Millipore Corporations	MAB354
Thyroid Transcription Factor 1 (NKX2.1) 1:500	Novocastra	NCL-L-TTR-1

**Bacterial and Virus strains**		

pGP-AAV1-syn-jGCaMP7f-WPRE (or comparable AAV)	Addgene	104488-AAV1

**Biological samples**		

Mouse Embryonic Fibroblasts (MEFs)	Millipore Sigma	PMEF-CF-K

**Chemicals, peptides, and recombinant proteins**		

Cytiva Hyclone Dulbecco’s High Glucose Modified Eagles Medium (DMEM High Glucose)	Gibco	SH30022.02
Gibco™ B-27™ Supplement (50×), minus vitamin A	Gibco	12-587-010
Dispase 5U/mL	Stem Cell Technologies	07913
Embryonic Stem Cell FBS	Gibco	10-439-024
GlutaMAX (100×)	Gibco	35-050-061
Penicillin-Streptomycin (Pen/Strep) (100×)	Gibco	15-140-122
DMEM/F12	Gibco	11-320-082
KnockOut Serum Replacement (KSR)	Gibco	10828028
MEM Non-Essential Amino Acids Solution (NEAA) (100×)	Gibco	11-140-076
InviPrimocin-Antimicrobial agent (500×)	InvivoGen	NC9392943
2-Mrcaptoethanol (2-ME) (0.1mM)	Gibco	21985023
Deoxyribonuclease I (DNASE)	Worthington Biochem	LK003172
Y27632 (ROCK Inhibitor)	Biopioneer	SM-008
Trypsin Inhibitor from Glycine Max	Sigma Aldrich	501784529
Trypsin-EDTA (0.05%) phenol Red	Gibco	25300054
Glasgow’s MEM (GMEM)	Gibco	11710035
1mM Pyruvate	Gibco	11360070
Smoothened Agonist,SAG	MilliporeSigma	56-666-1500UG
N-2 Supplement (100×)	Gibco	17502048
Chemically Defined Lipid Concentration (CDLC)	Gibco	11905031
Methylcellulose, viscotiy 15cP	ThermoScientific Chemicals	182312500
CHIR 99021	Fisher Scientific	442310
Human BMP4 Recombinant Protein	Gibco	120-05-50UG
Matrigel GFR Basement Membrane Matrix	Corning	CB-40230A
Human LIF Recombinant Protein	Gibco	300–05
Neurobasal Medium	Gibco	21-103-049
Phosphate-Buffered Saline (DPBS, 1×), Dulbecco’s formula, without calcium, without magnesium	ThermoScientific Chemicals	AAJ67802AP
EmbryoMax Primary Mouse Embryonic Fibroblasts (MEFs)	Sigma Aldrich	PMEM-CF
EmbryoMax 0.1% Gelatin Solution	Millipore Sigma	ES-006-B
Trypan Blue Solution, 0.4%	Gibco	15-250-061
FGF2 DISCs – 10ng/mL	Stemculture	NC2135529
HEPES	Sigma Aldrich Fine Chemicals Biosciences	NC0229916
Potassium Chloride (KCl)	Fisher BioReagents	BP366 500
Magnesium Chloride Hexahydrate (MgCl2)	Fisher Chemical	M33-500
Calcium Chloride Dihydrate (Cacl2)	Fisher Chemical	C79-500
Sodium Chloride (NaCl)	Fisher BioReagents	BP358-1
Glucose	Gibco	A2494001
Sodium Pyruvate (White Powder)	Fisher BioReagents	BP356 100
Sodium Dihydrogen Phosphate	FisherBrand	S381-500
L-Glutamine, Cell Culture Reagent	ThermoScientific Chemicals	AAJ6057314
Phosphate-buffered saline (DPBS, 1×), Dulbecco’s formula	ThermoFisher Chemicals	AAJ67802AP
16% Paraformaldehyde, Ted Pella Inc	Fisher	NC1537886
Andwin Scientific Tissue-Tek™ CRYO-OCT Compound	Fisher	1437365
D-Sucrose	Fisher	210513

**Experimental models: Cell lines**		

hiPSC lines	Provided by Dr. Jack Parent,^3^ University of Michigan	

**Deposited data**		

Original data	This paper	Zenodo: https://zenodo.org/records/18763819

**Software and algorithms**		

Leica Application Suite X (LAS X) v3.7	Leica Microsystems	RRID:SCR_013673; https://www.leica-microsystems.com/products/microscope-software/p/leica-las-x-ls/
Fiji v2.14.0	Schindelin, et al., 2012^4^	RRID:SCR_002285; https://fiji.sc/
ImageJ v1.54f (included with Fiji)	Schneider, et al., 2012^5^	RRID:SCR_003070;https://imagej.nih.gov/ij/
MATLAB R2024b	The MathWorks, Inc.	RRID:SCR_001622; https://www.mathworks.com/products/matlab
Calcium Imaging Data Analysis (CaImAn)	Giovannucci, et al., 2019^6^	RRID:SCR_021533; https://github.com/flatironinstitute/CaImAn
NormCorre	Pnevmatikakis & Giovannucci, 2017^7^	https://github.com/flatironinstitute/NoRMCorre
EZcalcium	Cai, et al., 2016^8^	https://github.com/jounhong/ezCalcium
CVX: MATLAB Software for Disciplined Convex Programming	CVX Research, Inc.; Grant & Boyd, 2009^9^	http://cvxr.com/cvx/
STAR_Protoc 1.0.0	This paper	Zenodo: https://zenodo.org/records/18763819

**Other**		

StemPro EZPassage Tool	Invitrogen	23181–010
Sterile Cell Strainer 100 μM Nylon Mesh	FisherBrand	22363549
CELLSTAR 6 Well Cell Culture Plate sterile, with lid	Greiner Bio-One	657160
PrimeSurface® 3D culture: Ultra-low Attachment Plates: 96 well, V bottom, Clear plates	S-Bio	MS-9096VZ
lumox® multiwell, Cell culture plate, with foil base, 24 well	Sarstedt	94.6110.024
Corning™ Centrifuge Tubes with CentriStar™ Cap 15 mL	Corning	430790
Corning™ Centrifuge Tubes with CentriStar™ Cap 50 mL	Corning	430828
Fisherbrand™ Sterile Polystyrene Disposable Serological Pipets with Magnifier Stripe 5 mL	Fisherbrand	13-678-11D
Fisherbrand™ Sterile Polystyrene Disposable Serological Pipets with Magnifier Stripe 10 mL	Fisherbrand	13-678-11E
Fisherbrand™ Sterile Polystyrene Disposable Serological Pipets with Magnifier Stripe 25 mL	Fisherbrand	13-678-11
Fisherbrand™ Sterile Polystyrene Disposable Serological Pipets with Magnifier Stripe 50 mL	Fisherbrand	13-678-11F
Fisherbrand™ Borosilicate Glass Disposable Serological Pipets with Regular Tip, Short Length 10 mL	Fisherbrand	13-678-36C

## Materials and equipment

**CRITICAL:** Materials purchased in bulk, namely Mouse-Embryonic Fibroblasts (MEFs), N-2 Supplement, Knockout Serum Replacement (KSR), and B27 without Vitamin A, are tested for quality control across different media batches to determine which batch is most suitable for organoids before purchase. To assess quality, organoids are treated with these materials and subsequently stained for cortical development markers, including, but not limited to, FOXG1, EMX1/2, SP8, COUP-TF1, CTIP2, and SATB2. The batch with the best development is then purchased in bulk and used until testing needs to be repeated for the next batch.

Vacuum filter media (0.22 μm pore size) in a culture hood. Add Matrigel and Methylcellulose directly to the media. Keep media sterile afterwards for up to 1 month at 4°C. Make 50 mL aliquots as needed each week to prewarm in a 37°C bead bath before use. Only add small molecules to 50 mL aliquots. Stem cells and 96-well plates are kept in 5% CO2 incubators, while organoids in petri dishes are kept in a multi-gas incubator 37°C, 40% O_2_, 5% CO_2_.

**Table:** CF1-Mouse Embryonic Fibroblasts (MEF) media

Reagent	Final concentration	Amount
DMEM High Glucose	N/A	440 mL
FBS	10% (v/v)	50 mL
Glutamax (100×)	1×	5 mL
Pen/Strep (100×)	1×	5 mL
**Total**	**N/A**	**500mL**

Store at 4°C for up to 1 month.

**Table:** Human embryonic stem cell (hESC) media for induced pluripotent stem cells (iPSCs)

Reagent	Final concentration	Amount
DMEM/F12	N/A	388 mL
20% KSR	4% v/v	100 mL
NEAA (100×)	1×	5 mL
Glutamax (100×)	1×	5 mL
Primocin (500×)	1×	1 mL
2-ME (0.1mM)	1×	908 μL
**Total**	**N/A**	**500 mL**

Store at 4°C for up to 1 month.

**Table:** Trypsin solution for organoid generation

Reagent	Final concentration	Amount
Trypsin solution	1×	948 uL
DNASE	0.05mg/mL	50 uL
ROCK inhibitor Y-27632	30 uM	3 uL
**Total**	**N/A**	**∼1 mL**

Store at 4°C for up to 1 month.

**CRITICAL:** Adjust amount prepared based on number of cell lines used for organoid generation.

**Table:** Trypsin inhibitor solution for organoid generation

Reagent	Final concentration	Amount
Trypsin Inhibitor from Glycine Max	1×	998 uL
ROCKi	30 uM	3 uL
**Total**	**N/A**	**∼1 mL**

Store at 4°C for up to 1 month.

**CRITICAL:** Adjust based on number of independent cell lines used for organoid generation. One cell line requires 1 mL.

**Table:** Sasai - Cortical Differentiation (SASCD) Medium for organoid maturation and neural induction

Reagent	Final concentration	Amount
GMEM	N/A	388 mL
KSR	20% (v/v)	100 mL
0.1mM NEAA	1×	5mL
1mM Pyruvate	1×	5mL
0.1mM 2-ME	1×	908 uL
Primocin	1×	1 mL
**Total**	**N/A**	**500mL**

Store at 4°C for up to 1 month.

**Table:** SASCD + ROCKi (‘Y’) medium for organoid neural induction

Reagent	Final concentration	Amount
GMEM	N/A	388 mL
KSR	20% (v/v)	100 mL
0.1mM NEAA	1×	5mL
1mM Pyruvate	1×	5mL
0.1mM 2-ME	1×	908 uL
Primocin	1×	1 mL
ROCKI	20 uM	100 uL/50 mL
**Total**	**N/A**	**500mL**

Store at 4°C for up to 1 month.

**Table:** SASCD + SAG (sonic hedgehog smooth agonist) given to organoids undergoing ganglionic eminence (GE) differentiation on D15

Reagent	Final concentration	Amount
GMEM	N/A	388 mL
KSR	20% (v/v)	100 mL
0.1mM NEAA	1×	5mL
1mM Pyruvate	1×	5mL
0.1mM 2-ME	1×	908 uL
Primocin	1×	1 mL
SAG	1 uM	1 μL/10mL
**Total**	**N/A**	**500mL**

Store at 4°C for up to 1 month.

**Table:** N2SASAI+SAG medium for ganglionic eminence differentiation on D18

Reagent	Final concentration	Amount
DMEM/F12	N/A	458 mL
N-2 Supplement	1×	5 mL
CDLC	1×	5 mL
Glutamax	1×	5 mL
Primocin	1×	1mL
8.5 % Methylcellulose	0.442% (w/v)	26 mL
SAG	1 uM	1 μL/10mL
**Total**	**N/A**	**500mL**

Store at 4°C for up to 1 month.

**Table:** 10% FBS + BMP4 + CHIR medium for early hippocampal (Hc) differentiation in organoids on D18

Reagent	Final concentration	Amount
DMEM/F12	N/A	400 mL
ES-qualified FBS	10% (v/v)	50 mL
Glutamax	1×	5 mL
N-2 Supplement	1×	5 mL
Chemically Defined Lipid Concentrate (CDLC)	1×	5 mL
Primocin	1×	1 mL
8.5 % Methylcellulose	0.442% (w/v)	26 mL
CHIR	3 uM	1.2 μL/4 mL
BMP4	0.5 nM	10 μL/4mL
**Total**	**N/A**	**500mL**

Store at 4°C for up to 1 month.

**Table:** N2-SASAI medium given to D18-D35 Cortical and D21 – D35 GE organoids, to ensure neural development

Reagent	Final concentration	Amount
DMEM/F12	N/A	458 mL
N-2 Supplement	1×	5 mL
Chemically Defined Lipid Concentrate (CDLC)	1×	5 mL
Glutamax	1×	5 mL
Primocin	1×	1mL
8.5 % Methylcellulose	0.442% (w/v)	26 mL
**Total**	**N/A**	**500mL**

Store at 4°C for up to 1 month.

**Table:** 10% FBS medium given to Hc organoids on D21-D42 for further differential development

Reagent	Final concentration	Amount
DMEM/F12	N/A	400 mL
ES FBS	10% (v/v)	50 mL
Glutamax	1×	5 mL
N-2 Supplement	1×	5 mL
Chemically Defined Lipid Concentrate (CDLC)	1×	5 mL
Primocin	1×	1 mL
Methylcellulose	0.442% (w/v)	26 mL

Store at 4°C for up to 1 month.

**Table:** 8.5% Methylcellulose stock solution: N2B27 and neurobasal media additive to prevent organoid merging (before the actual fusion of different kinds of organoids)

Reagent	Final concentration	Amount
Methylcellulose powder	8.5 %	20g (40mL of powder in 50 mL conical tube ×2)
Heated DMEM/F12 – 32 sec	N/A	74 mL
Cold DMEM/F12	N/A	165 mL
**Total**	**N/A**	**500mL**

Store at 4°C for up to 1 month.

**Table:** N2B27 + hlif medium given Cx and GE organoids +D35 and Cx+GE assembloids for further neural maturation

Reagent	Final concentration	Amount
DMEM/F12	N/A	458 mL
N-2 Supplement	1×	5 mL
B-27 Supplement without VA	1×	10 mL
Chemically Defined Lipid Concentrate (CDLC)	1×	5 mL
Glutamax	1×	5 mL
Primocin	1×	1 mL
8.5 % Methylcellulose	0.442% (w/v)	26 mL
Matrigel (GFR)	1% (v/v)	5 mL
Human LIF	5 μg/mL	10 μL/50 mL
**Total**	**N/A**	**500mL**

Store at 4°C for up to 1 month.

**CRITICAL:** Only add 10 μL Human LIF to 50 mL aliquots, not directly to stock media.

**Table:** Neurobasal + hlif medium given to Hc organoids +D42 and Hc+GE assembloids for further neural maturation

Reagent	Final concentration	Amount
Neurobasal Medium	N/A	399 mL
ES FBS	10% (v/v)	50 mL
Chemically Defined Lipid Concentrate (CDLC)	1×	5 mL
Glutamax	1×	5 mL
B-27 Supplement	1×	10 mL
N-2 Supplement	1×	5 mL
Primocin	1×	1 mL
Methyllcellulose	1% (v/v)	25 mL
Human LIF	5 μg/mL	10 μL/50 mL
**Total**	**N/A**	**500mL**

Store at 4°C for up to 1 month.

**CRITICAL:** Only add 10 μL Human LIF to 50 mL aliquots, not directly to stock media.

**Table:** 200× Ringer Solution for Artificial Cerebral Spinal Fluid

Reagent	Final concentration	Amount
NaCl	–	73.6g
Glucose	–	18.0g
Sodium Pyruvate	–	1.56g
Sodium Dihydrogen Phosphaste	–	1.72g
L Glutamine	–	1.46g
**Total**	**N/A**	**N/A**

Store at 4°C for up to 1 month.

• In a 2L beaker, add a stirring bar and place it on a magnetic stirrer plate, then turn it on.
  ○ While the stir plate is running, add the materials.
  ○ Once mixed, test the pH; the desired pH is 7.35–7.40.
  ○ If the solution is too acidic, add a small amount of NaOH and measure again.
  ○ If the solution is too basic, add a small amount of HCl and measure again.
    - Repeat this process until the desired pH is achieved.
• Add DI water to the solution until the volume reaches 500 mL.

**Table:** Artificial Cerebral Spinal Fluid (aCSF)

Reagent	Final concentration	Amount
520 mM Hepes	240 mM	50 mL
Ringer Solution (Prepared)	N/A	50 mL
1M KCl	46.2 mM	5000 uL
1M MgCl2	11.1 mM	1200 uL
1M CaCl2	18.5 mM	2000 uL
**Total**	**N/A**	–

Store at 4°C for up to 1 month.

• In a 2L beaker, add a stirring bar and place it on a magnetic stirrer plate, then turn it on.
• Add DI water to the solution until the volume reaches 1000 mL.
• Mix until all solvents are dissolved.
• Before the recording session, add 5 μL kainic acid for every 500 mL aCSF being used.

**CRITICAL:** Once kainic acid is added, the aCSF media can only be used for ∼24 h.

**Table:** 4% Paraformaldehyde Solution

Reagent	Final concentration	Amount
Paraformaldehyde (PFA) 16%	4% (v/v)	10 mL
dPBS	N/A	30mL
**Total**	**N/A**	**40 mL**

Store at 4°C for up to 1 week.

• In a chemical hood, break off the top of the PFA bottle.
• Use a P1000 to pipette the full contents of the PFA into the conical tube containing dPBS.

**Table:** 30% Sucrose Solution

Reagent	Final concentration	Amount
D-Sucrose	30% (v/v)	30 g
PBS	N/A	100 mL
**Total**	**N/A**	–

Store at 4°C for up to 1 month.

• Place a large beaker with a stir bar onto a stir plate.
• Add 100 mL PBS to the beaker.
• Measure out 30 g sucrose and pour into PBS.
• Allow stirring to continue until all the sucrose is dissolved.

## Step-by-step method details

### Mouse embryonic fibroblast plating

**Timing: 1 h for plating**

This section outlines the step-by-step preparation of a MEF feeder layer essential for culturing feeder-dependent hiPSCs.

***Note:*** MEF plating should be performed 3 days prior to stem cell passaging. MEF concentration should be optimized for each lot. This is an example of one lot we optimized for cortical organoid differentiation. All steps must be performed using aseptic technique in a sterile tissue culture hood to prevent contamination.

1. Prepare MEF aliquots:
  a. 9 mL in a 15 mL conical tube, 32 mL, and 35 mL in separate 50 mL conical tubes.
2. Prepare 6-Well Plates (1 MEF vial = 5 full plates, or 30 wells):
  a. Add 1 mL of EmbryoMax Gelatin Solution to each well.
    i. Tap each side of the plate 5 times to ensure the whole well and its sides are coated.
    ii. Leave on for 10–15 min at room temperature.
    iii. Aspirate gelatin from each well and add 1 mL of MEF media from the 35 mL aliquot to each well.
3. Prepare MEFs for plating:
  a. Thaw one frozen MEF vial in a hot water bath until a small amount of ice remains.
  b. Drop-wise, add 1 mL of MEF media into the MEF vial. With each drop, gently swirl the vial to mix it to avoid osmotic shock.
  c. Transfer the MEFs + media mixture to the 15mL conical tube carefully with no bubbles.
  d. Drop-wise, add 5mL of MEF media. After a couple of drops, gently swirl the tube to avoid osmotic shock. Directly (not drop-wise) add another 4 mL of MEF media and gently mix the entire tube.
  e. Wash the inside of the MEF vial a final time with 1 mL of MEF media.
  f. Centrifuge MEFs + media mixture at 188G for 5 min.
4. Plate MEFs onto 6-Well Plates:
  a. Gently aspirate the media after centrifuging until only the pellet remains.
  b. Transfer 5 mL of media from the 32 mL aliquot.
    i. Gently break up the pellet with a 5 mL serological pipette.
      ***Note:*** Be careful to avoid bubbles.
    ii. Resuspend in the remaining 31 mL of media.
  c. Add 1100 μL of the MEF mixture to each well dropwise and place in an incubator.
  d. To make sure equal distribution of the MEF in each well, for one hour after the plating, the plate should not be moved, and the incubator should not be shaken.
  e. Two days later, change the media in each well with 2 mL hESC media.
    ***Note:*** The following day (3 days post-plating), MEFs are ready for stem cells to be plated on.

### Stem cell passaging

**Timing: 1 h per full 6-well plate**

***Note:*** Passaging should be performed weekly and timed to be 3 days after MEF plating.

This section outlines the mechanical passaging of feeder-dependent hiPSCs to maintain and expand the culture. The process involves assessing colony confluency, dislodging differentiated colonies, mechanically dissociating the colonies from their plate, and replating the resulting suspension onto a previously prepared MEF feeder layer.^10^^,^^11^ Passaging is done on the same day each week, while cell suspension dilutions are adjusted based on cell density and confluency. The wells in which stem cells are passaged also contain fibroblast growth layer 2 (FGF2) discs to maintain consistent fibroblast growth factor signaling, promoting pluripotency and self-renewal while reducing spontaneous differentiation. This approach decreases feeding frequency by stabilizing FGF2 availability, improving culture consistency and efficiency. Note: Wells of hiPSC colonies from the previous week are divided, with a portion used for routine passaging to maintain the cell line and the remainder harvested for plating to initiate organoid generation. All steps must be performed using aseptic technique in a sterile tissue culture hood to prevent contamination. The passage dilution and timing should be optimized for each hPSC line.

5. Pre-warm three 50-mL aliquots of hESC media in a 37°C bead bath for 30 min.
6. Prepare the MEF plates for hiPSC transfer:
  a. Use a 2 mL aspirating pipette attached to a P200 pipette tip to aspirate the media in each well of the 6-Well plate.
    i. Wash wells with dPBS twice to remove any MEF media.
    ii. Add 2mL of pre-warmed hESC media to each well.
    iii. Add 1 FGF disc to each well.
  b. Mark the line and passage number on each plate.

***Note:*** Passage numbers increase by one each time the cells are passaged. If you passage cells that have been previously frozen down and thawed, increase the labeled passage number by one.

7. Prepare the stem cells:
  a. Under a microscope, observe the confluency of the stem cells in each well.
    i. Determine the dilution factor based on this.

***Note:*** To maintain the observed confluency, use a dilution ratio of 1:6, with wells of cells to mL of hESC media, respectively.; Over-confluent cells should be reduced with a greater dilution ratio e.g. 1:8.; Under-confluent cells undergo less dilution and/or should be passaged with more than one well e.g. 1:3–1:4.

**Figure 1:**
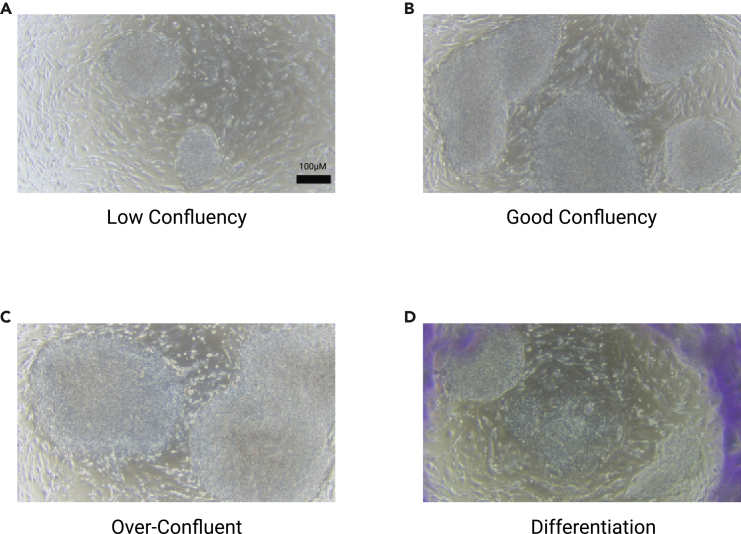
Examples of stem cell confluency (A) Examples of stem cells at under confluency, ideal confluency (B), over confluency (C), and differentiation (D) Under confluent cells appear sparse across the entire well and should be passaged at a lower dilution (less than 6 mL). Stem cells at an ideal confluency appear large and round throughout the well’s surface and should be maintained by having a cell suspension of 6 mL when passaged. Over confluent cells appear in small, clumped colonies and should be passaged at a higher dilution (greater than 6 mL). Scale bar is 100 μM.

8. Scratch differentiated stem cells.
  ***Note:*** Refer to Figure 1 to assess stem cell viability.
  a. Use a P200 pipette tip under a microscope in a TC hood to remove the differentiated portion before changing media.
    ***Note:*** If scratching is required more than once a week due to high concentrations of differentiated cell, this indicates unhealthy stem cells (see troubleshooting problem 1).
9. Prepare tools:
  a. Prepare EZ Passage tools.
    ***Note:*** Use a separate passage tool for each line/condition of stem cells to prevent cross-contamination.
    i. Manually submerge and agitate the tools in 100% ethanol 4 times and leave them to dry in the hood without touching any surfaces.
    ii. Rinse with dPBS before use to wash away remaining ethanol and maintain a stable isotonic environment for cells.
  b. Label 15 mL conical tubes so that each tube corresponds to a specific line/condition of stem cells undergoing passaging.
10. Collect cells.
  a. Carefully aspirate media in the 6-well plates.
    i. Add 3 mL of pre-warmed hESC media to each well.
  b. Gently move the passage tool up and down diagonally four times to cut hESC colonies.
  c. Rotate the plate 90 degrees and repeat to further cut hESC colonies.
  d. Move your passage tool to the outer edge of the well and turn the plate 2 times.
    i. Repeat this for each plate twice, waiting ∼1 minute before repeating the same plate.
  e. Clean the passage tools with 100% ethanol and let air dry.
11. Transfer cells from 6-well plate.
  a. Pre-wet a 10 mL glass pipette with hESC media.
  b. Use the pipette carefully to pick up all the media and freshly cut stem cell colonies.
  c. Release the media in a “rainbow-like” (semi-circular) motion on the top of the well to detach small colonies.
    i. Repeat the motion for the middle and bottom of the well.
    ii. Pick up all the media and transfer to the corresponding conical tube.
  d. Repeat for every well being passaged.
  e. Pipette 4 mL of hESC media and dispense it into the well, applying force to detach any remaining colonies.
    ***Note:*** Only use a glass pipette as using a P1000 pipette tip will produce small/unhealthy hESC colonies.
    i. Use this same method to passage all wells.
  f. Dispense all cells and media into the respective conical tubes.
  g. Centrifuge conical tubes at 150G for 2 min.
12. Resuspend cells.
  a. Aspirate the media until you reach the pellet and then add the predetermined amount of hESC media for dilution.
  b. Use a P1000 to break apart the pellet – maximum 10× trituration.
13. Transfer cells to fresh MEF plate.
  a. In the prepared MEF plate, add 1000 μL of hESC media and cell mixture to each well dropwise.
    i. Pipette up and down 3× between each well to ensure even distribution.
  b. Once all wells contain cells, gently move the 6-well plate up and down, side to side, and in a figure-eight motion.
    i. Repeat these motions eight to ensure even cell distribution.
  c. Keep it in the TC incubator (37°C, 5% CO_2_).

### Stem cell maintenance

**Timing: 30 min preparation, 5 min per plate**

Protocol for hiPSCs media changes.

***Note:* T**hese steps are performed on days 2 and 4 after passaging. Media change steps must be performed using aseptic technique in a sterile tissue culture hood to prevent contamination.

14. Prewarm 50-mL hESC media aliquots in a 37°C bead bath for 30 min.
15. Change media:
  a. Carefully aspirate the media, avoiding contact with the surface of the 6-Well plate and the FGF disc. Note: If the disc becomes visibly damaged, replace it with a new one.
  b. Replace with 3 mL fresh hESC media in each well.
  c. Place plates back into the incubator.

### Organoid generation—Plating

**Timing: 1.5 h per iPSC line**

This section details the process by which hiPSCs are used to generate embryoid bodies and subsequently brain organoids. To ensure standardized sizes of the resulting organoids, quantification of viable cells is performed using a hemocytometer prior to the final plating step, as described below.^1^^,^^12^

***Note:*** Wells of hiPSC colonies from the previous week are divided, with a portion used for routine passaging to maintain the cell line and the remainder harvested for plating to initiate organoid generation. All steps must be performed using aseptic technique in a sterile tissue culture hood to prevent contamination. Plating is performed weekly.

16. Prepare cells:
  a. In the remaining wells from the 6-well plate, add 3 μL ROCKi per well.
    i. Allow incubation for more than 30 min in the cell culture incubator at 37°C.
17. Prepare materials and media.
  a. Media: trypsin solution, trypsin inhibitor, dispase, hESC media, and SASCD + Y.
    i. Use 2, 50 mL aliquots of hESC media – dependent on the number of lines being plated.
      ***Note:*** Prepare 1 mL more for trypsin/trypsin inhibitor than the number of lines being plated. E.g., If two lines are being plated, prepare 3 mL.
    ii. Prepare 10 mL of SASCD+Y/v96-well plate.
      ***Note:*** The amount of SASCD +Y needed is dependent on the number of plates being used for organoid generation. E.g., 2 plates = 20 mL SASCD+Y.
    iii. All media should be prewarmed in a 37°C bead bath for 30 min.
  b. Prepare 96-Well plates:
    i. Use a multi-channel pipette to dispense 100 μL sterile dPBS in each well to wash it.
    ii. Aspirate and label with date, line, passage number, and batch number.
      ***Note:*** Batch numbers start at one and increase each time plating is done.
18. Collect and transfer cells from 6-Well plates:
  a. Aspirate hESC media from the 6-Well plates, including the FGF discs used during passaging.
    i. Add 2 mL sterile dPBS to each well to maintain the physiological environment of the cells.
    ii. Aspirate again, add 500 μL dispase to each well, and incubate at 37°C to dissociate cells. Leave on for about two min.
      ***Note:*** Make sure that colonies start to detach and then aspirate.
  b. Add 3 mL hESC media to each well.
    i. Use a pre-wet glass pipette and release media in “rainbow-like” (semi-circular) motion, as done during passaging.
    ii. Carefully dispense all collected cell colonies into a 15 mL conical tube.
    iii. Repeat across all wells.
  c. Use 4 mL of hESC media to dislodge the remaining colonies in the well.
    i. Move the 4 mL to the next well and repeat.
    ii. After all cells have been collected, dispense into the conical tube.
      ***Note:*** If colonies are still attached to the well, use a P1000 pipette to apply force to pick them up and dispense them.
  d. Set the conical tube aside for 2 min to allow cell colonies to settle to the bottom and the MEFs still floating in the media to separate from them.
    i. Aspirate the media until 2 mL is reached to remove most of the MEFs and resuspend with 8 mL of hESC media.
  e. Centrifuge the conical tube at 150G for 2 min.
19. Dissociate cells.
  a. Aspirate the media carefully and resuspend in 1 mL of trypsin solution.
    i. Gently tap the tube for 2 min to break up the pellet and allow the trypsin solution to mix.
  b. After 2 min, add 1 mL trypsin inhibitor solution.
    i. Break up the cells using manual force with a P1000 pipette.
      ***Note:*** No bubbles should be created.
  c. Use a pre-wetted glass pipette to dispense 8 mL of hESC media into a conical tube.
    i. Place a sterile 100 μm cell strainer onto a 50 mL conical tube.
    ii. Pick up the 10 mL media and cell mixture from the conical tube and dispense through the filter to remove big cell clumps.
  d. Dispense cells back into the original 15 mL conical tube.
  e. Centrifuge the cells at 95 G for 2 min.
20. Calculate the quantity of cells for plating.
  a. Aspirate the media until the pellet and resuspend with 1 mL SASCD+Y media.
    i. Use a P1000 to break up the pellet very gently and without generating bubbles.
  b. In a sterile Eppendorf tube, mix 10 μL Trypan Blue with 10 μL of the cell suspension and media solution.
    i. Add 10 μL of this mixture to a hemocytometer with a coverslip.
    ii. Under a microscope, count the cells in two 4-by-4 squares of the hemocytometer.

***Note:*** A square corresponds to 1 × 10^−4^ mL volume Sections being counted from should be on opposite diagonals of the hemocytometer to ensure accurate and representative cell counting.

21. Perform final plating.
  a. Calculate the amount of cell solution to dispense into SASCD+Y so that the final number of cells per each well will be 9000 cells in 100 μL.
    i. Y = sum of two 4-by-4 squares.
    ii. (Y/2) × 2 dilution factor = # of cells per 1 × 10^−4^ mL.
    iii. Y cells/1 × 10^−4^ mL = Y × 10^4^ cells/mL.
    iv. For 10 mL, 900,000 cells are needed.
    v. 900,000 cells/(Y × 10 cells/mL) = Z mL cell suspension needed for 10 mL (1 plate).
    vi. To save time, we just do 90/Y = Z uL cell suspension needed for 10mL.
  b. In a 50-mL conical tube, combine the SASCD+Y media with the calculated volume of cells to achieve a final volume of 10 mL.
  c. Pour the 10 mL solution into a reagent reservoir.
    i. Dispense 100 μL of the cell solution with a multichannel pipette into the preprepared 96-well plate.
  d. Centrifuge at 200G for 2 min.
  e. Place into the incubator (37°C, 5% CO_2_).

### Organoid differentiation/maintenance: Cortical, ganglionic-eminence, and hippocampal fates

**Timing: <1 h per day for maintenance of neural induction, transfer to Petri dishes, and maintenance of differentiated organoids**

This protocol outlines the process of guiding nascent organoids toward three distinct neural regional fates: cortical (Cx), ganglionic eminence (GE), and hippocampal (Hc). Following a period of neural induction by inhibiting TGFβ and WNT signaling (in our approach, by passive induction in minimal media and NOT with dual SMAD inhibition), the organoids first adopt a dorsal forebrain (cortical) fate. However, other desired regional identities can be achieved through the timed application of specific morphogens – smoothened agonist (SAG) for a ventral (GE) fate, a combination of bone morphogenetic protein (BMP4) and WNT agonist CHIR 99021 for dorsal midline fates, including the hippocampus.^1^^,^^11^^,^^12^ Organoids are transferred to a new, multi-gas incubator on D18 to reduce hypoxia-induced cell death, improve oxygenation of the organoid core to reduce central necrosis, and mimic in vivo oxygen levels. On D35, Cx and GE organoids are cut, while Hc organoids are cut on D42. This is done to release the necrotic center and enhance oxygen and nutrient transfer.^4^ Organoids are typically ∼3.8mm in diameter at this point, while they reach ∼4.1mm on D56.

***Note:*** All steps must be performed using aseptic technique in a sterile tissue culture hood to prevent contamination.

**Figure 2:**
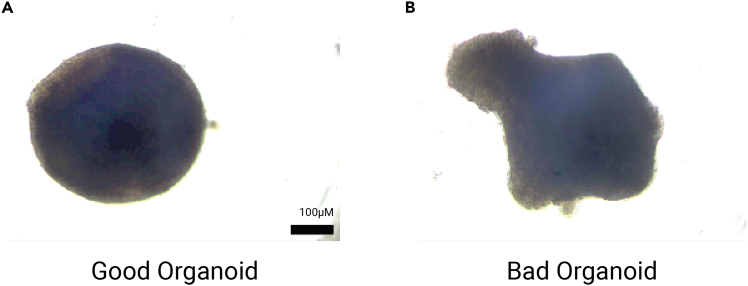
Examplles of good and bad organoids (A) Examples of a good organoid and a bad organoid (B). Good organoids will appear round and well-formed, with a clear epithelial layer seen around the edges. Rosettes are sometimes formed in early organoid stages and will appear as a circular cluster of elongated cells arranged radially. Incorrectly formed organoids show no clear epithelial layer or rosettes and often look misshapen – appearing more as a clump of cells rather than a clear organoid. Scale bar is 100 μM.

**Figure 3:**
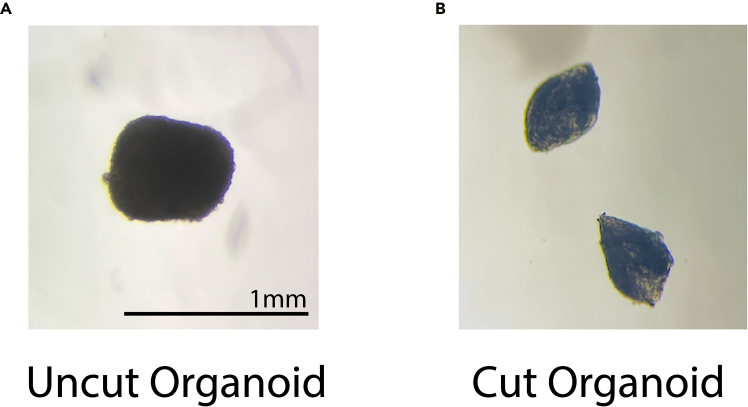
Representative images of organoids before and after cutting (A) Representative images of organoids before and after (B) cutting. Organoids are cut in half with minimal damage to the tissue in order to release the necrotic core and ensure proper cellular maturation. Scale bar is 1mm.

22. Neural induction of organoids.
  a. Three days (D3) after plating, dispense 100μL SASCD+Y media to all wells in the 96-well plate.
  b. On D6, remove 120 μL media and add 80 μL SASCD media.
  c. On D9, remove 75 μL of media and add 80 μL of SASCD into each well.
    i. Repeat this on D12.
      ***Note:*** Do not touch the bottom of the wells so that the organoids aren’t picked up.
  d. On D15, remove 75 μL of media from each well.
    i. Mark 1/3 to 1/2 of the plate to indicate SASCD+SAG [1 μM] has been added to ensure ganglionic-eminence (GE) fates.
    ii. Add 80 μL SASCD+SAG to these wells.
    iii. Add 80 μL SASCD-Y to the rest of the wells to continue cortical (Cx) and hippocampal (Hc) differentiation.
23. Transfer organoids to petri dishes for further development.
  a. On D18, determine which organoids have developed enough to be transferred for further growth and differentiation.
    i. Look under a microscope and mark those that form correctly; discard the others.
      ***Note:*** Reference Figure 2 to determine successful organoid development; Correctly formed organoids will be round, with a clear epithelial layer along the edges. Rosettes sometimes form at this point, appearing as a circular structure of elongated cells; Incorrectly formed organoids show no clear epithelial layer, rosettes, or shape (see troubleshooting problem 2).
  b. Prepare 3 sterile petri dishes per line.
    i. Label each with line, batch number, date, and organoid fate (Cx, GE, or hippocampus).
  c. Use wide-bore pipette tips and transfer organoids treated with SAG to the GE-labeled dish.
  d. Transfer the remaining organoids for cortical and hippocampal fate specification – equal amounts in each dish, or change based on experimental needs.
  e. Remove old media carefully from the 6-cm petri dishes with a P1000 pipette.
  f. Add 4 mL of media to each dish, respective of desired organoid fates. See composition in materials and equipment.
    i. GE – N2SASAI+SAG [1 μM].
    ii. Cx – N2SASAI.
    iii. Hc – FBS + BMP4 [0.98 nm] + CHIR [3.0 μM].
  g. Transfer plates to a multi-gas incubator (37°C, 40% O_2_, 5% CO_2_).
24. On D21, aspirate old media in the petri dishes and add 4 mL of preheated, fresh media.
  a. Cx and GE – N2SASAI.
  b. Hc – FBS.
    i. Treat organoids with this media until D35 for Cx and GE, D42 for Hc.
  c. Change media again on D23, D25, D28, D30, etc. (i.e., MWF of every week).
25. Remove necrotic centers (Cutting).
  a. Prepare 50mL of N2B27 + hLIF for D35 Cx and GE organoids and neurobasal + hLIF [0.10 nM] for D42 Hc organoids to continue maturation.
  b. Transfer D35 (Cx), D35 GE, and D42 Hc organoids from the incubator to sterile hood with a microscope.
  c. Carefully use surgical microscissors to cut the organoids in half.
    ***Note:*** Organoids should appear with minimal damage following the cutting process (see Figure 3). If the organoid is severely damaged during cutting, i.e., falling apart or multiple deep slices, discard (reference troubleshoot problem 3).
  d. Aspirate the old media and add 4mL of media for each respective organoid fate.
    **CRITICAL:** On D35 onward, change Cx and GE organoids with N2B27 media rather than N2SASAI; On D42 onward, change Hc organoids with neurobasal media. These media should be used to change the old media after cutting.

### Assembloid generation from single organoids

**Timing: 15 min per Petri dish for cutting and fusion; 5 min per Petri dish for transfers; 10 min per plate for ongoing assembloid maintenance**

**Figure 4:**
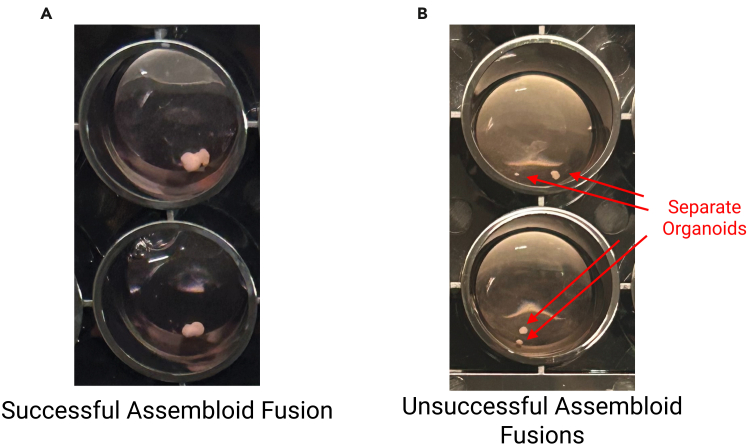
Examples of fusion outcomes (A) Unsuccessful fusions (B). Successful fusions are firmly stuck together and do not fray or separate. Unsuccessful fusions appear as two separate organoids and/or are not firmly bound together. They may also appear frayed.

The following provides a step-by-step protocol for generating and maintaining cortical (Cx+GE) and hippocampal (Hc+GE) assembloids. Compared to organoids, assembloids better recapitulate the cellular composition and circuit complexity developing brain and are well-suited for studies involving interneuron migratory defects,^2^ neurodevelopmental disorders, and network dysfunction.^1^^,^^13^ Examples of successful and unsuccessful fusions are shown in Figure 4.

***Note:*** All steps must be performed using aseptic technique in a sterile tissue culture hood to prevent contamination.

26. Create assembloids (D56) (Fusing).
  a. Cut organoids in half as done when removing necrotic tissue.
  b. Use a P1000 with the tip cut off and transfer Cx and Hc organoids to sterile Eppendorf tubes.
    ***Note:*** Only one organoid should be placed in each tube.
  c. Transfer GE organoids so that each tube has either both Cx and GE, or Hc and GE.
  d. Aspirate old media add 400μL media to each tube.
    i. Give Eppendorf’s containing Cx and GE N2B27 + hlif media.
    ii. Give Eppendorf’s containing Hc and GE neurobasal +hlif.
  e. Use parafilm to cover the tops of the Eppendorf tubes rather than closing them to allow for oxygenation.
    i. Poke 4 to 5 small holes in the parafilm using a needle.
  f. Transfer the Eppendorf tubes back to the original organoid incubator and incubate for 3 days in an upright and stationary position to ensure fusion.
    ***Note:*** Eppendorf tubes containing organoids for fusing should be kept upright in the incubator for the duration of the 3 days.
27. Transfer assembloids (D59).
  a. Prepare a hood with a 50 mL aliquot N2B27 + hlif, 50mL aliquot of neurobasal + hlif, and 24-Well lumox plates.
  b. Use a P1000 with the tip cut off and transfer one fused assembloid to each well.
    ***Note:*** Do not puncture lumox wells. If punctured, use a new plate.
  c. Remove old media and add 400μL media.
    i. Cx and GE fusions are given N2B27 + hlif.
    ii. Hc and GE fusions are given neurobasal +hlif.
      ***Note:*** If assembloids fail to fuse, discard them (see troubleshooting problem 4).
28. Change the media of the assembloids with 400μL of media the next day (D60).
29. Assembloid maintenance/media changes.
  a. Change the media of the assembloids 3× a week with 400μL of media.

***Note:*** Remember to warm the media up to 37°C prior to use and media is changed every other day. E.g.: D63, D65, D67, D70, D72, D74, etc. (Monday, Wednesday, Friday of every week).

**CRITICAL:** If assembloids ‘unfuse’, i.e. break apart, discard them (see troubleshooting problem 4).

### Quality control testing of media

**Timing: Up to 1 h for each fixing and blocking, performed over 2 sequential days; 1 h for sectioning; 1 h for imaging after completion of antibody treatment**

Materials purchased in bulk, namely Mouse-Embryonic Fibroblasts (MEFs), Knockout Serum Replacement (KSR), and N-2 Supplement are tested for quality control across different media batches to determine which batch is most suitable for organoids before purchase. To assess quality, organoids are treated with these materials and subsequently stained for cortical (Cx) development markers, including, but not limited to, FOXG1, EMX1/2, SP8, COUP-TF1, CTIP2, and SATB2. The batch with the best development is then purchased in bulk and used until testing needs to be repeated for the next batch. General quality control testing of organoid development varies by fate. Cx organoids can be stained for the markers listed above, while hippocampal (Hc) organoids can be stained for EMX2, PROX1, FOXG1, CTIP2, SP8, COUP-TF1 and LHX2 markers, and ganglionic eminence (GE) organoids can be stained for FOXG1, NKX2.1, SST, and CTIP2 at different time points of development.

***Note:*** Quality control testing of media additives is not done on Hc or GE organoids, as the additives that tend to vary in efficacy are only added to N2SASAI/N2B27. Though GE organoids use this media, Cx organoids are the typical subject for this testing. The listed cell markers indicate whether Hc/GE fates are developing correctly.

30. Mouse-Embryonic Fibroblasts (MEF) testing on hiPSCs.
  a. Plate MEFs as described above so that each 6-well plate corresponds to a different batch of MEFs.
    i. The MEF batch being used as a control is the current one in use.
  b. Thaw and passage hiPSCs onto each plate as described previously.
  c. Generate organoids as described in plating and differentiate the organoids into cortical fate.
    i. Organoids should be maintained as usual.
  d. On D35, fix and section organoids for comparison.
31. Knockout Serum Replacement (KSR) testing.
  a. Prepare different batches of media containing KSR, including SASCD and SASCD+Y.
    i. The control KSR is the current one in use.
    ii. Note: SASCD+SAG does not have to be prepared in different batches of KSR, as only cortical organoids are used for testing.
  b. Plate organoids as described above, using SASCD+Y made from different KSR lots at the final step.
    ***Note:*** Ensure correct labeling to keep track of which organoids correspond to each batch.
  c. Change media with corresponding batches of SASCD +Y and SASCD as usual.
    ***Note:*** All other media should remain consistent (i.e., N2SASAI and N2B27).
  d. On D35, fix, block, and section cortical organoids that underwent KSR testing.
32. N-2 Supplement testing on cortical organoids.
  a. Prepare different batches of media for Cx organoids containing N-2 supplement.
    ***Note:*** Each batch corresponds to the lot numbers being investigated for use: the control N2 batch is the current batch in use, which includes N2SASAI media, and N2B27 media.
  b. On D18, when cortical organoids are transferred from 96w plates to petri dishes, separate Cx organoids into different dishes corresponding to the batch being used to test for development.
    i. Change media Monday, Wednesday, and Friday, as usual, continuing with the correct batch.
      ***Note:*** Media for Cx organoids switches from N2SASAI to N2B27 on D35, following the typical cutting procedure.
  c. On D56, rather than fusing, Cx organoids treated with different N-2 supplement are fixed.
33. Organoid Fixing.
  a. Transfer organoids from petri dishes to Eppendorf tubes using a P1000 pipette with the tip cut off.
  b. Wash each organoid with dPBS 3 times.
  c. Treat washed organoids with 4% paraformaldehyde in a chemical hood.
    i. Store the Eppendorf tubes in the 4°C for 20 min.
  d. Remove organoids from 4°C and wash again with dPBS 3 times.
  e. Remove all dPBS from tubes and add 1000 μL 30% sucrose solution.
    i. Place the Eppendorf tubes back in 4°C overnight.
34. Organoid Blocking.
  a. The day after fixing, remove organoids from the Eppendorf tubes and transfer them onto a petri dish using a P1000 with the tip cut off.
    i. Remove any remaining liquid surrounding the organoid to ensure the organoid is dry.
  b. Place a generous amount of Tissue-Tek O.C.T compound on top of the organoid and in the cryomold so that half the mold is filled.
  c. Use a P1000 with the tip cut off and carefully transfer the organoid from the petri dish to the cryomold.
    i. The Tissue-Tek placed on the organoid helps to hold and lubricate the organoid for this transfer.
    ii. Label the cryomold with the organoid type, batch number being tested, age, and line.
    iii. Place in −80°C overnight.
35. Organoid Sectioning.
  a. Pop the contents of the cryomold out, and use Tissue-Tek to attach the mold to the cryostat disc.
    i. Place disc into cryostat and allow the disc and tissue to freeze together using the cryobar setting.
  b. Set the section thickness to 16 μm to maintain cellular morphology and structural integrity for confocal imaging.
  c. When the cryobar is complete, attach the disc to the object holder.
  d. Adjust the blade position using the arrows until the blade is right ahead of the tissue.
  e. Carefully section the tissue, collecting six sections per slide.
    i. Continue sectioning until 3 to 4 slides contain good sections.
      ***Note:*** Good sections appear to have no big tears or holes in the tissue.
  f. Repeat this process for all the organoids that are being imaged for quality.
36. Treat organoids with antibodies for quality markers.
  a. This includes, but is not limited to:
    i. FOXG1, EMX1/2, SP8, COUP-TF1, CTIP2, and SATB2 for cortical organoids.
    ii. FOXG1, LHX2, EMX2, CTIP2, SP8, COUP-TF1, and PROX-1 for hippocampal organoids.
    iii. FOXG1, NKX2.1, SST, and CTIP2 for ganglionic eminence organoids.

***Note:*** Organoids are also stained with DAPI as a nuclear counterstain.

37. Image organoids to assess architectural structures.
  a. Compare organoid structures using antibody markers and assess which lots correspond to the most successful capitulations of cortical organoid architecture.

***Note:*** Good cortical architecture is described as follows: FOXG1 appears nuclear and abundant across rosettes, EMX1/2 is present at rosette cores, Rosettes appear with CTIP2 and SATB2 expression layered outside, CTIP2+ neurons appear deeper (less superficial) compared to SATB2 and CTIP2 is typically expressed earlier, SP8/COUP-TF2 shows regional gradients/patches across the organoid, showing patterning. Successful hippocampal fate is as follows: FOXG1 and LHX2 are localized to the core of rosettes, EMX2 and COUP-TF1 expressed in progenitors will appear surrounding the rosette’s center, CTIP2 will appear sparse and scattered in the peripheral neuronal layer, PROX1 will appear as distinct dentate-like clusters. Ganglionic Eminence organoid fate architecture is described as follows: FOXG1 and NKX2.1 expression are central to ventricular rosettes, CTIP2-expressing cells will appear adjacent to the ventricular rosettes, SST+ neurons will appear outside NKX2.1+ rosettes, often appearing to extend radially.

**CRITICAL:** Ensure staining specificity. Evaluate antibody labeling in relation to DAPI nuclear staining, confirming that the signal is confined to cellular regions. A lack of staining specificity typically indicates a poor tissue/organoid quality.

38. Physiological Analysis of organoids for cellular activity are done to further determine quality.

***Note:*** An example of physiological analysis includes calculating calcium spike number and total number of active neurons using calcium indicator imaging (see below) in D120 assembloids. The data will be compared with assembloids generated at the same time from the gold-standard media components (ideal) or normative data, as determined by prior experiments.

### Adeno-associated virus infection of cortical and hippocampal assembloids

**Timing: 5 min per assembloid, repeated after 24 and 48 h**

These steps outline how we use an adeno-associated virus (AAV) to deliver the gene for a genetically encoded calcium indicator to generate assembloids, enabling optical imaging of their neural activity. Current commercially available vectors have been specifically designed for this purpose. For example, pGP-AAV1-*syn*-jGCaMP7f-WPRE (Addgene 104488-AAV1) employs a human synapsin (syn) promoter to restrict jGCaMP7f expression to neurons with efficient transduction of neural cells. A successful infection allows for the visualization of calcium dynamics, a reliable proxy for neural firing, and thus facilitates the characterization of spatiotemporal network activity within the assembloid. If no GFP signal or activity is observed, please refer to troubleshooting problems 5 and 6 below.

39. Infect cortical or hippocampal assembloids (age ∼D106) with a genetically encoded calcium indicator using AAV vector (such as pGP-AAV1-*syn*-jGCaMP7f-WPRE).
  a. Transfer assembloids to a separate 24-well lumox plate for virus treatment.
    i. Add ∼9.9×10^10^ genome copies (GC) in a total of 250 μL media.
      ***Note:*** Cortical assembloids recieve N2B27 + hlif, while hippocampal assembloids recieve neurobasal + hlif media N2B27 + hlif for cortical assembloids.
    ii. Return to incubator (37°C, 40% O_2_, 5% CO_2_).
      ***Note:*** The high O_2_ concentration drives diffusion through the organoids with the lumox plates allowing optimal gas exchange.
  b. After 24hr, add 750 μL media to each previously treated well.
    ***Note:*** Do not aspirate the prior 250 μL media.
  c. After another 24hr, remove all 1000 μL from the virus-treated wells and replace with 400 μL of the appropriate media.
    i. Continue the typical schedule of assembloid media changes until ∼D120; 14 days post-AAV-treatment.

### Perform two-photon imaging

**Timing: 30 min for setup and ∼1 h per assembloid**

This protocol details a method for capturing functional network dynamics in assembloids using 2P calcium imaging. Key steps include Matrigel immobilization of an individual assembloid to reduce movement during the recording and perfusion with warmed aCSF containing kainic acid (as is standard practice^14^). The setup and acquisition parameters are optimized for Leica multiphoton systems running LAS X software (but are broadly applicable to other multiphoton systems). The resulting videos captured from multiple regions of the assembloid can be subsequently processed to analyze the functional state of the assembloid neuronal network.

**Figure 5:**
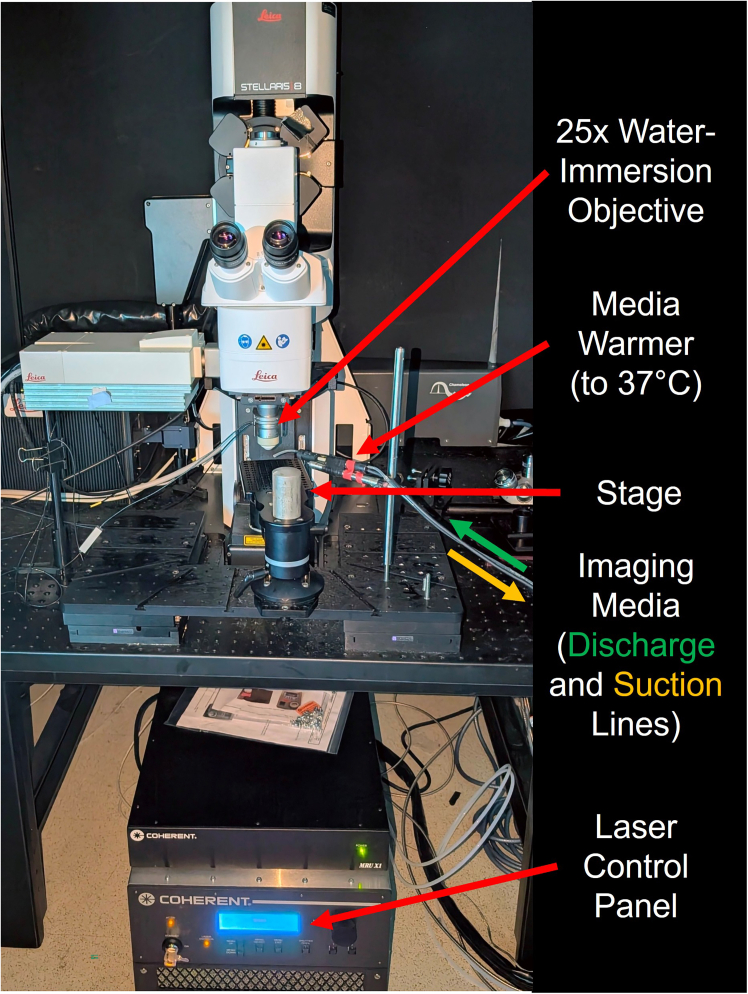
Diagram showing the setup of our Leica multiphoton system used for 2P image acquisition of AAV-treated assembloids

**Figure 6:**
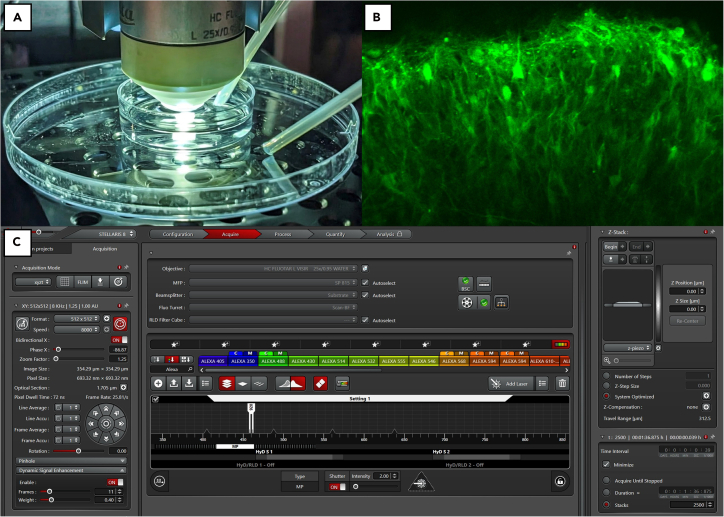
2-photon microscope set-up (A) Brightfield microscopy sets the initial FOV (assembloid in center of dish) prior to 2P. Temperature is maintained in the dish through continuous flow of warmed imaging media via inlet and outlet tubing. Video frame (B) captured during 2P imaging demonstrating an appropriate FOV with multiple fluorescing neurons along the edge of the assembloid. Typical LAS X interface (C) with settings used for acquisition.

40. Sample preparation: Approximately 14 days after AAV treatment, prepare each assembloid for imaging.
  a. Mount the assembloid by transferring a single assembloid to the center of a 35×10mm polystyrene petri dish.
    i. Carefully pipette away all excess media.
  b. Apply 10 μL of cold (4°C) phenol red-free Matrigel to the base of the assembloid for embedding.
  c. Incubate the assembloid by placing the lid on the petri dish and return it to the incubator (37°C) for 5 min to allow the Matrigel to solidify.
41. Configure the system for live imaging.
  ***Note:***Figure 5 shows the gross system setup. Figure 6A shows the petri dish with embedded assembloid and imaging media perfusion. Figure 6B shows a representative example of the desired field-of-view for assembloid 2P image acquisition. Figure 6C shows the typical acquisition parameters of the LAS X software.
  a. Perfusion: Remove the petri dish from the incubator and place on the microscope stage.
    i. Begin perfusing the dish with warmed (37°C) artificial cerebral spinal fluid (aCSF) solution containing kainic acid (1:100,000 v/v).
    ii. Ensure the perfusion rate maintains the sample’s temperature at 37°C.
      ***Note:*** A typical recording requires ∼300 mL of aCSF.
  b. Laser Configuration: On the Leica application suite (LAS X), power on the laser and tune it to 920 nm (or the appropriate wavelength for your fluorophore).
    ***Note:*** Our tested setup includes a Leica Stellaris multiphoton system equipped with Coherent Chameleon Ultra II tunable infrared laser.
  c. Locate Assembloid: Using the 25× water-immersion objective, position the lens ∼5mm above the assembloid.
    i. Switch to brightfield to locate the assembloid, adjusting the XY stage and focus as needed.
      ***Note:*** Search for the edge of the assembloid, which will typically have a faint blue-green color distinct from the background.
  d. Software Settings: In the LAS X software, set the desired acquisition parameters.
    ***Note:*** For example, the parameters can be set as follows: Resolution: 512×512 pixels. Scan Speed: 8000 Hz achieved using a resonance scanner (and set to bidirectional to further increase the frame rate); note that Lower scan speeds may reduce the accuracy of automated ROI detection and subsequent processing steps. Time Interval: Minimize, such that to capture 2500 frames it takes ∼96.5 s (∼25.9 Hz frame rate).
42. Image Acquisition: Record neuronal activity from optimal regions. Again, Figure 6B shows an example of optimal field-of-view for assembloid 2P image acquisition.
43. Find a Field-of-View (FOV).
  a. Activate the HyD RLD detector and begin scanning.
  b. Adjust the focal depth, XY position, gain, and laser intensity to find a region with clearly identifiable fluorescent neurons.

***Note:*** Using the minimal laser intensity possible avoids damage (burns) to the tissue. We set gain between 25%–30% and laser intensity between 10%–12% during typical usage.

44. Optimal Regions: Focus on the outer/superficial layers of the assembloid.

***Note:*** Superficial layers tend to have more mature and more active neurons. Select an FOV with a high density of distinct cell bodies to aid in later analysis. The ideal cell has a more brightly fluorescent periphery and a dimmer center. Cells that are diffusely bright may be dead/inactive.

45. Record data once an optimal FOV is selected.

***Note:*** Do not adjust the gain, laser intensity, or FOV during the acquisition.

46. Repeat and Save: Perform recordings in multiple regions to capture a comprehensive dataset for the assembloid. When finished, save all acquisitions as a single compressed Leica Image File (.lif) in the designated project folder.

### Pre-processing using ImageJ

**Timing: 10 min per Leica-formatted project file (containing multiple videos)**

This protocol outlines the pre-processing of two-photon imaging data using Fiji/ImageJ. Raw.lif (Leica) project files are imported, temporally downsampled to optimize subsequent processing, and auto-adjusted for brightness and contrast. The resulting videos are saved as individual.tiff files, making them ready for analysis in MATLAB. This entire workflow can be automated with a macro or performed manually for each video.

47. Import Video File: Open your Leica-formatted (.lif) project file in ImageJ.
  a. Drag and drop the desired.lif file into the main ImageJ window.
  b. The Bio-Formats Import Options window will open. Use the following settings:
    i. Under View stack with, make sure that Hyperstack is selected.
    ii. In the file list, select the specific videos you need. Never choose versions with “dse” (Dynamic Signal Enhancement), a proprietary Leica temporal smoothing approach, as these data are already partially down sampled.
    iii. Ensure 2D planar is selected.
  c. Click OK to import the video.
48. Temporally Downsample the Video: This step reduces the number of frames (temporal resolution) to make the file smaller and processing much faster and more accurate.
  a. Navigate to the menu bar and select Image > Adjust > Size and reduce depth by a factor of 10.
    ***Note:*** This is optimized for 25.8 Hz original frame rate – the extent of downsampling should be determined by your recording frame rate.
  b. Check Constrain aspect ratio and Average when downsizing and interpolation=Bilinear.
49. Adjust Brightness & Contrast: Optimize the video’s contrast for better visualization.
  a. From the menu, open Image > Adjust > Brightness/Contrast…
  b. Click the Auto button and then Apply.
50. Save as a Tiff File:
  a. Navigate to File > Save As > Tiff…
51. To process all videos within your.lif project file, you can repeat the above steps for each file.

***Note:*** These steps can be automated using the following ImageJ macro:

// --- START OF MACRO ---

var output_dir = “C:/Users/SLab/Desktop/My_Tiff_Results/”; //for Windows

if (nImages == 0) {exit(“Error: No images are open. Please open an image first.”);}

if (!File.exists(output_dir)) {File.makeDirectory(output_dir);}

print(“Starting preprocessing…”);

run(“Size...”, “width=512 height=512 depth=250 constrain average interpolation=Bilinear”);

run(“Enhance Contrast”, “saturated=0.35”);

run(“Apply LUT”, “stack”);

print(“Preprocessing complete.”);

var title = getTitle();

var output_path = output_dir + title + “_processed.tif”;

print(“Saving to: ” + output_path);

saveAs(“Tiff”, output_path);

print(“Save complete. ”);

// --- END OF MACRO ---

### Processing using EZcalcium in MATLAB

**Timing: 5**–**10 min per video, shorter when performed in batches**

**Figure 7:**
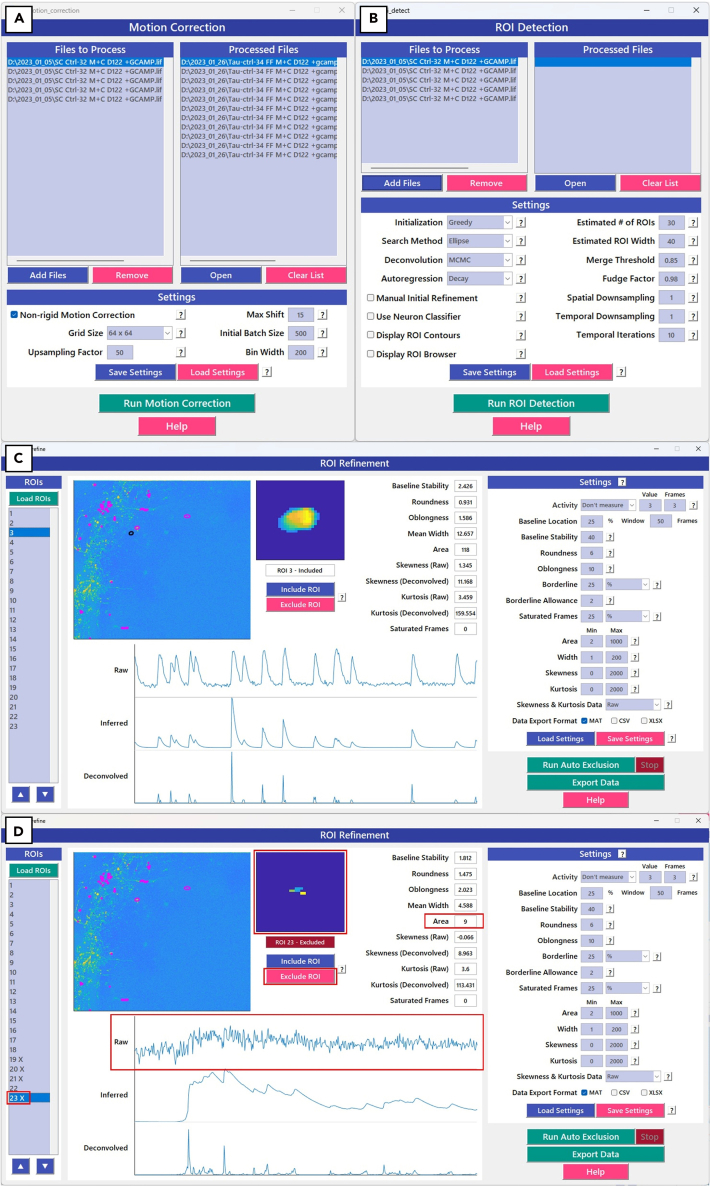
EZ calcium file processing and refinement for post 2-photon-based network analyses (A) EZcalcium motion correction^7^^,^^8^ and ROI detection (B) panels with typical settings. An example (C) of a good ROI during refinement with correctly-identified cell body and clear activity peaks in the “Raw” calcium transients; this ROI should be included in subsequent analysis. An example (D) of a bad ROI during refinement without a clear cell body identified and extremely small total area as well as lacking clear activity peaks in the “Raw” calcium transients (marked in red boxes); clicking “Exclude ROI” will add an “X” next to its number in the list of ROIs and will exclude it from the saved data when exported.

This protocol details the data processing workflow using the EZcalcium^8^ GUI in MATLAB. After ensuring all required toolboxes (EZcalcium, CaImAn,^6^ NoRMCorre,^7^ CVX^9^) are on the MATLAB path, the workflow proceeds in three main stages. Pre-processed.tiff files first undergo motion correction to correct for drift. Next, automated ROI detection identifies potential neurons, which are then manually refined by the user. The curated data is saved for subsequent analysis. While the settings recommended below are optimized for our typical workflow, you may need to adjust the parameters for optimal video processing. If these processing steps do not yield reasonable ROIs, please refer to troubleshooting problem 7 below. Extensive details on all parameters are available in the EZcalcium documentation (https://github.com/porteralab/EZcalcium). Examples from real data created using the parameters shown are provided in Figure 7. An example 2P video (STAR_Protoc_example_tif.zip) is available at https://github.com/samarasinghelab/STAR_Protoc that can be used to test the workflow and parameter choices and compare results with the corresponding refined ROI data (STAR_Protoc_example_mat.mat).

52. Open MATLAB and ensure that all subfolders of EZcalcium, CaImAn (included with EZcalcium), NoRMCorre (included with EZcalcium), and CVX toolboxes are available through the search path.
  a. Type the following: >ezcalcium.
53. From the main GUI:
  a. Motion Correction:
    i. Add Files that you recently pre-processed and saved through ImageJ.
    ii. Run Motion Correction.
    iii. Note: It is typically not necessary to change the default settings here.
  b. Automated ROI Detection.
    i. Use Add Files for the motion-corrected Tiff files (file name suffix of “_mcor”) and run ROI Detection with following settings.
    ii. Set Initialization: Greedy.
    iii. Set Search Method: Ellipse.
    iv. Set Deconvolution: MCMC (high-precision fully-Bayesian method).
    v. Set Autoregression: Decay (estimate only calcium indicator decay kinetics as this is less likely to overfit the downsampled data).
      ***Note:*** Estimated # ROIs should be set based on the number of neurons estimated in your FOV. Unless automated ROI detection performs poorly on your dataset, the other settings can be left at their default values.
  c. ROI Refinement.
    i. Use Load ROIs for each of the automated ROI detection results.
    ii. Manually Include or Exclude ROIs based on their activity profile and shape and then press Export Data.
      ***Note:*** Good ROIs should have clear peaks in their activity distinct from background and ideally represent cell bodies rather than axons. See Figure 7 for examples. Manual refinement does not require adjustment to the default settings.

### Network analysis

**Timing: 5 min per video**

**Figure 8:**
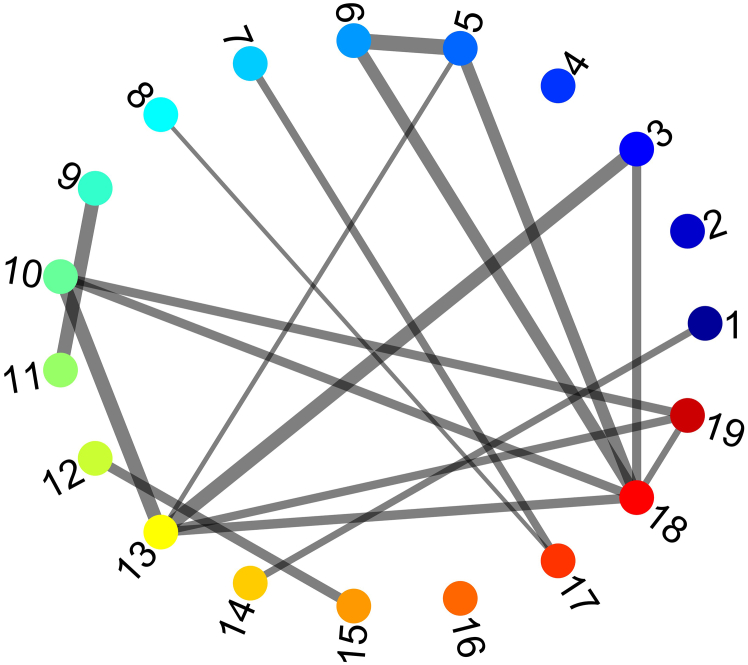
Representative functional connectivity network derived from assembloid 2P imaging The graph displays interactions between ROIs (neurons) 1–19, generated using the provided custom script. Edge thickness indicates the strength of functional connectivity based on cross-correlation values. These network topologies allow for further quantification using standard graph theoretic metrics (e.g., degree centrality, clustering coefficient, global efficiency).

Network analysis is the final step for characterizing assembloid networks from 2P imaging. This automated process uses a customizable MATLAB script (analyze_calcium_imaging..m at **Zenodo:** https://zenodo.org/records/18763819 (STAR_Protoc 1.0.0) to calculate key metrics like firing rates, burstiness, global synchrony, spectral coherence, and functional connectivity. An example network diagram of the functional connectivity is provided in Figure 8. The script appends all results directly to the processed.mat files, providing a quantitative foundation for subsequent plotting, statistical analysis, or further processing based on specific research goals. Refer to troubleshooting problem 8 if network analysis yields poor network results (such as low functional connectivity or synchronization). An example script (example_visualization.m) and accompanying refined ROI data (STAR_Protoc_example_mat.mat) are available at **Zenodo:** https://zenodo.org/records/18763819 (STAR_Protoc 1.0.0).

54. Open MATLAB and ensure that all subfolders of CaImAn^6^ and CVX^9^ toolboxes are available through the search path. Then in the folder containing analyze_calcium_imaging.m type: >analyze_calcium_imaging.
55. When prompted, select the desired previously processed.mat files to run network analysis.
  ***Note:*** Script parameters such as frame_period (default 0.3875s) and max_lag (default 13) should be set based on the temporal features of the recording and downsampling. The output from this analysis will be stored in the selected files. You can access or modify this output by simply loading the.mat files in matlab.
  a. Notable variables and their description are:
    i. burstiness: coefficient of variation of inter-spike interval for each ROI.
    ii. firing_rate_hz: firing rate in Hz for each ROI.
    iii. P_spike: spike probability matrix (ROIs × frames) where values can be >1 for multiple predicted spikes within that frame.
    iv. R: functional connectivity matrix (ROIs × ROIs, signed cross-correlation) with max_lag defined in the script parameter section. This can be used with R_significant to create a weighted adjacency matrix (Figure 8) for further network analysis.
    v. R_pvalue: p-values for each connection (via spike train shuffling with n_shuffles defined in the script parameter section).
    vi. R_significant: boolean mask for significant connections (based on significance_alpha level defined in the script parameter section).
    vii. global_synchrony_timeseries: population synchrony at each time point.
    viii. global_synchrony_index: average global synchrony across time.
    ix. coherence_matrix: spectral coherence matrix (ROIs × ROIs).
    x. spectral_coherence_index: average spectral coherence across all pairs.

## Expected outcomes

When performed correctly, this protocol will yield healthy hiPSC colonies, region-specific organoids, and stable cortical and hippocampal assembloids suitable for calcium-indicator imaging. hiPSCs should form round, compact, and firmly adherent colonies with minimal spontaneous differentiation. Organoids are expected to appear as spherical aggregates with a smooth outer epithelial layer and should remain structurally intact following cutting.

Successful cortical (Cx) organoid development, as assessed by immunohistochemistry, is characterized by robust expression of FOXG1 (∼D15), followed by dorsal telencephalic progenitor markers LHX2 and EMX1/2 (∼D25-30). Regional corticial patterning is evidenced by anterior SP8 and posterior COUP-TF1 expression (∼D30-40). This development is followed by the maturation of deep-layer CTIP2+ neurons (∼D50-60), which precedes the expression of SATB2+ neurons (∼D70-100+).

Hippocampal (Hc) organoid development is marked by early forebrain marker expression of FOXG1 (∼D15), and LHX2 and EMX2 expression ∼D20. Dentate gyrus-like identity is verified by robust PROX1 expression (∼D50-70). Low/transient cortical markers SP8 and COUP-TF1 may be present early on, while low CTIP2 expression (∼D56) is exhibited.

Ganglionic eminence (GE) organoids exhibit forebrain identity (FOXG1 ∼D15-D20), followed by ventral progenitor cell expression of NKX2.1 (∼D30-40). Inhibitory interneuron maturation is observed, indicative of SST+ cell expression (∼D80+). Low levels of CTIP2 may be present (∼D56), consistent with the emergence of striatal-like neuronal populations.

After fusion, organoids should adhere tightly and form stable assembloids without tearing or fraying. Over time, cortical and hippocampal organoids will exhibit visible integration with their GE counterparts. For example, by pre-treating GE organoids with CAG:tdTomato AAV, we previously observed widespread interneuron migration into the Cx and Hc compartments within the resulting assembloids.^1^ Fluorescence-based markers can confirm that mature assembloids recapitulate expected cellular development and organization. Mature Cx+GE exhibit cortical layer organization (TBR1/SATB2 expression) while mature Hc+GE display distinct regional identities (KA1/PROX1 expression). Both types contain mature inhibitory neurons (GAD65/SST).

Following successful AAV delivery of calcium indicators, 2-photon calcium imaging should reveal clear fluorescence transients corresponding to neuronal activity. These signals are expected to exhibit high signal-to-noise ratios, stable baselines, and measurable ΔF/F_0_ changes associated with spontaneous network events. MATLAB-based analysis will extract individual and population-level activity features, including event rates, synchrony measures, and network burst dynamics.

Collectively, users should expect robust assembloid formation and reproducible calcium-imaging data that support quantitative assessment of spatiotemporal network activity.

## Limitations

Assembloids offer access to human neural circuit organization but remain constrained by several inherent limitations. Although this protocol extends traditional organoid models by introducing greater interneuron diversity, the resulting tissues still represent early developmental stages and do not fully capture the maturation, synaptic complexity, or circuit architecture of the adult human brain. Although fusion reliably produces stable assembloids, the extent and timing of integration between organoids can vary, and not all fused pairs achieve uniform structural or functional coupling. The absence of a functional vasculature further restricts long-term growth and limits metabolic and physiological fidelity. Further, the formation of necrotic cores within the organoids can negatively impact development, thus altering cellular responses and maturation. Though we aim to mitigate these effects using a multi-gas incubator and cutting organoids at different maturation stages, it is important to note that this can hinder accurate modeling.

Calcium-indicator imaging provides a powerful readout of population dynamics but is limited by the spatial resolution, depth penetration, and signal kinetics of the chosen indicator. AAV transduction efficiency may differ across organoid types and depths, potentially yielding uneven labeling. Calcium signals measure intracellular calcium influx rather than direct membrane voltage and thus may not capture fast spiking or inhibitory synaptic activity with high fidelity.

As with all stem cell–derived systems, variability can arise from iPSC line differences, stem cell health, cutting and fusion techniques, and media preparation. These factors may influence the consistency of organoid morphology, fusion efficiency, and downstream functional readouts. Users should interpret results within these constraints and, when possible, validate findings using complementary approaches.

## Troubleshooting

### Problem 1

As referenced in Step 7, stem cells may show consistent, high-volume differentiation.

### Potential solution

This could be a result of overconfluency, inconsistent media changes, and/or poor MEF quality. Remove differentiated colonies by scratching more than once a week; do so on an as-needed basis before changing media. Ensure MEFs are healthy; consider testing different MEF batches to determine which works best.

### Problem 2

As referenced in Step 22, organoids fail to form spheres.

### Potential solution

This could result from incomplete single-cell suspension and/or low viability. Ensure cells are passed through the single-cell strainer carefully and efficiently. If the cells appear in multi-cell colonies during counting, ensure the pellet has been sufficiently broken up. If stem cells look unhealthy prior to plating, skip plating that week and focus on stem cell health.

### Problem 3

As referenced in Step 24, it may be difficult to successfully cut organoids, or the 3-D structure of organoids is damaged during the cutting process.

### Potential solution

This is likely a result of handling the cutting process too aggressively. Begin by making a small cut into the organoid and use that as a guide point as to where your cut should come from. Avoid making multiple, small cuts on different part of the organoid. Use two pairs of micro scissors – one to gently hold the organoid in place and the other to make the actual cut. Removing ∼1–2 mL of media, while ensuring the organoids are still fully submerged, can aid in preventing the organoid from moving around too much while cutting.

### Problem 4

As referenced in Steps 26 and 28, organoids may fail to ‘fuse’– indicating a failure to generate an assembloid – or break apart.

### Potential solution

This likely reflects poor organoid health, agitation during the fusion process (e.g., samples were moved during the 3-days period), or incomplete cutting before fusion, as cut sides tend to fuse better. If previously fused assembloids break apart, discard them. Going forward, ensure that the assembloid is being handled gently – don’t dispense media directly onto the assembloid to ensure it’s not met with force, avoid unnecessarily touching the assembloid.

### Problem 5

As referred to in Step 38, a lack of GFP signal may occur.

### Potential solution

Check viral titer, viral health (e.g., was AAV freeze-thawed more than once, and exposure length – at least 10 days).

### Problem 6

As referenced in Step 38, GCaMP may appear visible but no activity may accompany it.

### Potential solution

Check if the media is warm, check aCSF contents and make up (verify it was prepared correctly). If all cells are bright, there may be a high proportion of dead cells; the ideal cell is brighter along its edges and dimmer in the middle. If there is tissue burning during acquisition, turn down the laser power and, if necessary, turn up the gain.

### Problem 7

As referenced in Step 50, EZcalcium^8^ processing may not yield reasonable ROIs.

### Potential solution

First, verify the input video quality in Fiji/ImageJ. The.tiff files should show distinct cells with clear, visible calcium transients (with changes in activity) and a good signal-to-noise ratio. If the video quality is adequate, adjust the automated ROI Detection parameters. A common starting point is setting the “Estimated # of ROIs” (step 48.b) to accurately reflect the number of neurons in your video’s field of view. If the results are still poor, consult the EZcalcium documentation for guidance on other parameters (e.g., initialization, search methods, “Use Neuron Classifier”). Lastly, if automated detection continues to fail, try “Manual Initial Refinement” to select “good” ROIs to seed the algorithm, which can help guide the automated detection.

### Problem 8

As seen in Step 52, network analysis may yield low functional connectivity or poor synchronization.

### Potential solution

First, confirm that the frame_period parameter (step 50) in the analyze_calcium_imaging.m script exactly matches the actual sampling period (in seconds) of your downsampled recording. An incorrect value here will invalidate the temporal metrics. If the parameter is correct, the results may be biologically meaningful (i.e., the network is genuinely weakly correlated). To confirm this is not a processing artifact, perform a sensitivity analysis. Test if the results remain consistent when moderately adjusting other script parameters such as max_lag or the temporal smoothing window (set smooth_window>1).

## Resource availability

### Lead contact

Requests for further information should be directed to and will be fulfilled by the lead contact, Ranmal A. Samarasinghe (rsamarasinghe@mednet.ucla.edu).

### Technical contact

Technical questions on executing this protocol should be directed to and will be answered by the technical contact, Ranmal A. Samarasinghe (rsamarasinghe@mednet.ucla.edu).

### Materials availability

This study did not generate new unique reagents.

### Data and code availability

• The accession number for the original MATLAB code (analyze_calcium_imaging.m, network analysis steps 49-50) and example data reported in this paper is Zenodo: https://zenodo.org/records/18763819. All other software used is publicly available and cited in the key resources table. The original published data generated using the methods described here are available from the lead contact upon reasonable request.

## Acknowledgments

The authors acknowledge the work was funded by the 10.13039/100000002National Institutes of Health (grant no. 5K08NS119747 to R.A.S.), the 10.13039/100000002National Institutes of Health (grant nos. R01MH130061 and #R01DA051897 to B.G.N.), 10.13039/100002736CURE Epilepsy (grant no. 20244243 to C.M.M.), 10.13039/100002736CURE Epilepsy and the International SCN8A Alliance/Wishes for Elliott (grant no. 20204012 to R.A.S.), 10.13039/100000893Simons Foundation (grant no. 717153 to R.A.S.), 10.13039/100005595UCLA 10.13039/100007857Intellectual and Developmental Disabilities Research Center (grant no. P50HD103557 to R.A.S. and B.G.N.), 10.13039/100008623Broad Stem Cell Research Center (BSCRC) Innovation Award (award no. 49660 to R.A.S.), the In Memory of Christina Louise George Fund (to R.A.S.), and the Michael R. Bloomberg Revocable Trust to (R.A.S.). The authors thank Jessie E. Buth for her assistance in the early development of the organoid generation protocol and Drs. Peyman Golshani and Jianis Taxidis for their guidance on multiphoton imaging. The graphical abstract and figures were created using Biorender.com.

## Author contributions

A.R.G. and C.M.M. wrote the manuscript, with edits from D.T., B.G.N., M.W., and R.A.S. M.W., B.G.N., and R.A.S. developed the protocol for assembloid generation, as well as parts of the 2P analysis. A.R.G. contributed to the protocol for assembloid generation. C.M.M. and D.T. helped develop the 2P analysis protocol.

## Declaration of interests

The authors declare no competing interests.

## Declaration of generative AI and AI-assisted technologies in the writing process

During the preparation of this work, the authors used ChatGPT and Gemini in order to improve the grammar and word choice to enhance readability of this manuscript. After using these tools, the authors reviewed and edited the content as needed and take full responsibility for the content of the published article.
